# Does exercise enhance the benefits of nutritional support on the biochemical markers of nutrition, anthropometry, and body composition in hemodialysis patients? A systematic review

**DOI:** 10.3389/fnut.2024.1471455

**Published:** 2024-11-29

**Authors:** Raheleh Kamalzadeh Yazdi, Nima Radkhah, Alireza Ostadrahimi

**Affiliations:** ^1^Student Research Committee, Tabriz University of Medical Sciences, Tabriz, Iran; ^2^Nutrition Research Center, Department of Clinical Nutrition, School of Nutrition and Food Sciences, Tabriz University of Medical Sciences, Tabriz, Iran

**Keywords:** exercise, nutritional support, biochemical markers of nutrition, anthropometry, body composition, hemodialysis, protein-energy wasting

## Abstract

**Background:**

Exercise and nutritional support are effective strategies in hemodialysis patients who often face health issues like protein-energy wasting (PEW). Therefore, this study aimed to evaluate whether combining exercise with nutritional support offers additional benefits for anthropometry, body composition, and biochemical markers of nutrition in hemodialysis patients.

**Methods:**

This systematic review searched databases, including PubMed, Scopus, and Web of Science, until 14 February 2024 to identify relevant randomized controlled trials. Following screening and data extraction, quality assessment was conducted using the revised Cochrane Risk of Bias tool (ROB2). The study was reported following PRISMA guidelines.

**Results:**

Six studies comprising 199 male and female hemodialysis patients were included. These studies did not report any significant differences in anthropometry, body composition, and nutritional status between individuals who received an exercise program along with nutritional support and those who received only nutritional support.

**Conclusion:**

This systematic review suggests that the combination of exercise with nutritional support may not improve the positive effects of nutritional support on anthropometry, body composition, and nutritional status in hemodialysis patients. However, due to the low quality and significant heterogeneity among the existing studies, further research is required to draw definitive conclusions.

**Systematic review registration:**

PROSPERO (www.crd.york.ac.uk/prospero; registration no: CRD42024542613).

## 1 Introduction

End-stage renal disease (ESRD) is the final and permanent stage of chronic kidney disease, which occurs when the kidneys are no longer able to function well enough to support long-term survival without renal replacement therapy (1). Globally, its prevalence is projected to increase from 2.6 million in 2010 to 5.4 million in 2030 (2). Renal replacement therapy has different approaches, with hemodialysis being the most common choice for ESRD patients (3–5). However, it often causes protein-energy wasting (PEW), a syndrome defined by adverse nutrition and body composition changes leading to fat and muscle depletion (6). Hormonal imbalances, systemic inflammation, increased catabolism, the release of myocytokines, and the retention of toxins due to uremic syndrome are some factors contributing to decreased functionality and PEW (7). Various nutritional and body composition markers are used to identify PEW, such as hypoalbuminemia, a key biomarker and a strong predictor of mortality in this population (8–10). Unintentional weight loss or a decrease in BMI can also be important indicators of PEW (8). Chronic kidney disease (CKD), particularly in those undergoing dialysis, often leads to impaired physical function and physical inactivity (11). However, regular exercise has been shown to be beneficial in preventing and treating uremic muscle wasting, improving cardiac function, reducing cardiovascular risk factors, increasing muscular strength, and enhancing exercise capacity (12–14). The Kidney Disease Improving Global Outcomes (KDIGO) 2024 Guideline even recommends that CKD patients undertake moderate-intensity physical activity for at least 150 min a week, or to a level suited to their cardiovascular and physical tolerance (15). Olvera-Soto et al. (16) found that three-month resistance exercise significantly increased arm muscle area, arm muscle circumference, handgrip strength, and body fat percentage. In addition, Torres et al. (17) demonstrated that a combination of resistance training and flexibility exercise for 3 months improved body composition parameters (BMI and lean tissue index) and lipid profiles (total cholesterol, LDL, and triglycerides). In another study, Liao et al. (18) indicated that three-month cycling exercise significantly increased albumin levels and BMI and reduced inflammatory cytokines such as hs-CRP and IL-6. Similarly, optimal nutritional support has shown the potential to improve lean body mass or albumin levels (19, 20). Some studies also explored the effects of nutritional support in these patients. Qin et al. (21) observed that two-month oral nutritional supplementation significantly increased serum albumin, hemoglobin, and dietary energy intake among hemodialysis patients (21). Furthermore, a recent consensus statement suggests that the combination of adequate nutrition and exercise may be more effective than either intervention alone in preventing muscle loss among dialysis patients. However, this study also indicated that further investigation in clinical trials is necessary (22). Therefore, our study aims to systematically summarize randomized controlled trials that concentrated solely on hemodialysis patients to evaluate if the addition of exercise to nutritional support demonstrates any extra benefits on biochemical markers of nutrition, anthropometry, and body composition in this population.

## 2 Materials and methods

### 2.1 Study design

This systematic review was reported following the Preferred Reporting Items for Systematic Reviews and Meta-analysis (PRISMA) guidelines (23), with the protocol registered on PROSPERO (www.crd.york.ac.uk/prospero; registration no: CRD42024542613).

### 2.2 Search strategy

We systematically searched PubMed, Web of Science, and Scopus databases until 14 February 2024, using the following search strategy: (“Exercise” OR “Physical fitness” OR “Sports” OR “Exercise therapy” OR “Physical Activity” OR “Exercise training” OR “Physical exercise”) AND (“Nutritional support” OR “Nutritional supplementation” OR “Oral nutritional supplementation” OR “Nutritional intervention” OR “Nutritional therapy” OR “Enteral nutrition” OR “Parenteral nutrition”) AND (“Biomarkers of nutritional status” OR “Nutritional status indicators” OR “Nutritional status” OR “Nutritional assessment” OR “Nutritional biomarkers” OR “Malnutrition” OR “Biochemical indicators” OR “Biochemical parameters”) OR (“Body composition” OR “Body fat distribution” OR “Body fat percentage” OR “Lean body mass” OR LBM OR “Body weights and measures” OR “Body mass index” OR BMI OR “Anthropometry” OR “Skinfold thickness” OR “Waist-Hip ratio”; Supplementary Tables 1, 2). Moreover, the references of the retrieved articles and existing reviews were manually checked for additional resources.

### 2.3 Inclusion and exclusion criteria

We only included randomized controlled trials (RCTs) evaluating the effects of combining an exercise program with nutritional support on nutritional biochemical markers, anthropometric indices, and body composition in male and female hemodialysis patients undergoing dialysis at least twice a week. Only English-language articles, with no restrictions on publication date, were included. Moreover, we excluded studies other than RCTs, those lacking relevant data, and studies that involved individuals under the age of 18. In addition, we excluded RCTs investigating the sole effect of either exercise or nutritional support. We also excluded studies with a control group not receiving nutritional support. The PICO (Population, Intervention, Comparison, and Outcomes) framework is shown in Table 1.

**Table 1:** PICO for study inclusion.

Participants (P)	Intervention (I)	Comparison (C)	Outcomes (O)
Inclusion criteria: ^*^Hemodialysis patients ^*^≥18 years	Exercise program and nutritional support	Nutritional support	Serum albumin, creatinine, C-reactive protein (CRP), fat mass percentage, weight, body mass index (BMI), mid-arm circumference, arm muscle circumference, arm muscle area, or muscle strength
Exclusion criteria: ^*^Non-hemodialysis patients ^*^<18 years	RCTs focused solely on either exercise or nutritional support	Not receiving nutritional support	

RCTs, Randomized Controlled Trials.

### 2.4 Study screening

Two independent reviewers (RKY, NR) carefully reviewed all retrieved articles by reading their titles and abstracts. If there was any uncertainty about excluding a study, it was reviewed in full to reduce the risk of accidental exclusion. Two reviewers then independently analyzed the full texts of potentially relevant papers. Any disagreements were resolved by a third party (AO). We used a PRISMA flowchart to summarize this process.

### 2.5 Data extraction

The studies included in the analysis underwent a standardized data extraction process using a spreadsheet prepared by one of the authors (RKY). A second reviewer (NR) verified the extracted data to minimize errors and bias. If any data was missing from the reports, we made efforts to contact study authors to obtain necessary information. In cases where a study had more than two comparisons, only the ones meeting the eligibility criteria were considered. The following details were extracted: first author, publication year, study location, health status, age, gender, study design, sample size, type and protocol of exercise program, amount of nutritional support received, method of nutritional support administration in both intervention and control groups, duration of interventions, and the means and standard deviations or median (first and third quartiles) of the outcomes at baseline and post-intervention.

### 2.6 Quality assessment

Two reviewers (RKY, NR) independently assessed the quality of the articles using the Cochrane tool for assessing the risk of bias in randomized controlled trials (RoB 2) (24). This tool consists of seven domains: (I) random sequence generation; (II) allocation concealment; (III) blinding of participants and personnel; (IV) blinding of outcome assessment; (V) incomplete outcome data; (VI) selective reporting; and (VII) other sources of bias. Any disagreements were reconciled by a third party (AO). The risk of bias is presented in Table 2.

**Table 2:** Risk of bias assessed according to the revised cochrane risk-of-bias tool for randomized trials (RoB 2).

Study	Random sequence generation	Allocation concealment	Blinding (participants and personnel)	Blinding (outcome assessment)	Incomplete outcome data	Selective reporting	Other sources of bias	General risk of bias
Dong et al. (25)	Low	Unclear	Low	High	Unclear	Unclear	Low	high risk of bias
Hristea et al. (26)	Low	Low	Low	Unclear	Unclear	Low	Low	some concerns
Martin-Alemañy et al. (27)	Low	Unclear	Low	Unclear	Low	Unclear	Low	high risk of bias
Jeong et al. (28)	Low	Low	Low	Unclear	Low	Unclear	Low	some concerns
Martin-Alemañy et al. (29)	Low	Unclear	Low	Unclear	High	Unclear	Low	high risk of bias
Martin-Alemañy et al. (30)	Low	Unclear	Low	Unclear	Unclear	Unclear	Low	high risk of bias

### 2.7 Data synthesis

We extracted data from the included studies and presented it in table format. The study outcomes are outlined in the results section. Due to the limited number of studies and the high heterogeneity, we could not conduct a meta-analysis.

## 3 Results

### 3.1 Study selection and characteristics

**Figure 1:**
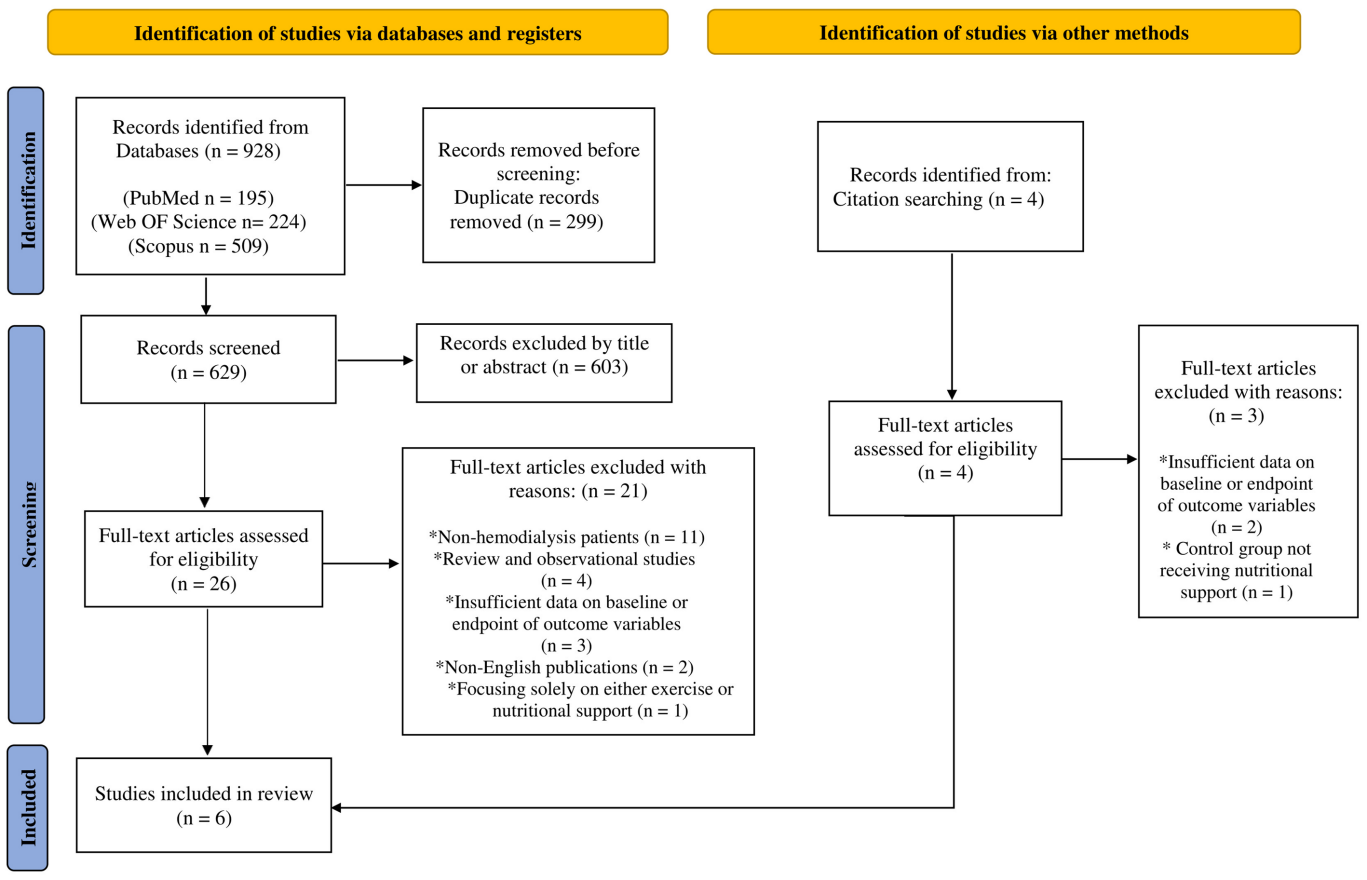
Flowchart of study selection for inclusion trials in the systematic review.

A total of 928 articles were discovered through a comprehensive search, and four more were found from reference lists. After removing duplicates, 629 papers were screened for eligibility by reviewing their titles and abstracts. Thirty articles were then assessed in full-text form, and 24 studies were excluded for the following reasons: (a) studies with non-hemodialysis population, (b) review and observational studies, (c) studies with insufficient data on baseline or endpoint of outcome variables, (d) non-English articles, (e) studies solely focused on either exercise or nutritional support, and (f) studies with a control group not receiving nutritional support. Ultimately, six articles, including two pilot studies, were included in the current review (25–30). The flow chart for the process of the study selection is displayed in Figure 1. A total of 199 male and female hemodialysis patients were included in the study. There were 94 individuals in the intervention group (exercise program and nutritional support) and 105 participants in the control group (nutritional support). All subjects received oral nutritional supplements (ONS) or intradialytic parenteral nutrition (IDPN) for nutritional support. The protein content of each can of ONS was between 16.7 and 20 grams. Those in the intervention groups also participated in resistance, aerobic, or a combination of both exercise programs. In addition, one study implemented the exercise program 30 min before hemodialysis (25), and other studies conducted it during dialysis (26–30). The duration of the studies varied from 3 to 12 months, and the mean age of the patients ranged from 29 to 70 years. All studies were published between 2011 and 2022. Three trials [Martin-Alemañy et al. (27); Martin-Alemañy et al. (29); and Martin-Alemañy et al. (30)] were conducted in Mexico, one trial [Hristea et al. (26)] was performed in France, and two trials [Dong et al. (25); and Jeong et al. (28)] were conducted in the USA. Studies by Hristea et al. (26) and Martin-Alemañy et al. (30) were pilot studies. The characteristics of the studies are presented in Table 3.

**Table 3:** The characteristics of the included randomized clinical trials.

Author, Year	Country	Design	Status	Mean ages (years)	Sample size	Male/Female (numbers)	Intervention (EX + NS)	Control (NS)	^*^Intervention dialysis Vintage (months)	^*^Control dialysis Vintage (months)	EX-Protocol	Quantity of NS ingested	Duration (months)
Dong et al. (25)	USA	RCT	Hemodialysis	43.2	22	NR	One group: Resistance Exercise+ IDON (n = 10)	IDON (n = 12)	> 3	> 3	3 sets of 12 repetitions of leg-press within 30 min before each hemodialysis session	2 cans of IDON (lactose-free formula) each can (240 mL and 480 kcal,16.7 gr protein, 52.8 gr carbohydrates, and 22.7 gr fat)	6
Hristea et al. (26)	France	RCT	Hemodialysis with PEW	69.7	16	NR	One group: Aerobic exercise + ONC or IDPN (if ONC intolerance) (n = 7)	ONC or IDPN (if ONC intolerance) (n = 9)	139 (255.5)	96 (86)	30 min individualized cycling on a cycloergometer at the beginning of hemodialysis	Adapted to patient needs based on the dietary record to achieve the goals set by the EBP guidelines for gender energy intake 30–40 kcal/kg of ideal weight/day, and protein intake >1.1 g/kg of ideal weight/day (34)	6
Martin-Alemañy et al. (27)	Mexico	RCT	Hemodialysis	34	36	(15) / (21)	One group: Resistance Exercise + ONS (n = 17)	ONS (n = 19)	NR	NR	4 sets of 30 repetitions of resistance exercises for 40 min during the second hour of hemodialysis	1 can of ONS (including high-oleic safflower oil, corn syrup solids and FOSs) (434 kcal, 19.2 gr protein and 22.8 gr lipids) (low in vitamins A and D and high in folates and vitamin B6)	3
Jeong et al. (28)	USA	RCT	Hemodialysis	54.8	67	NR	One group: Exercise + Whey protein (n = 29)	Whey protein (n = 38)	34.3 ± 34.8	45.6 ± 38.7	45 min cycling on cycle ergometer during each dialysis session	30 gram whey protein supplement at each dialysis session, mixed into 4–6 ounces of water	12
Martin-Alemañy et al. (29)	Mexico	RCT	Hemodialysis	29	34	NR	Two groups: ^*^Aerobic exercise + ONS (n = 12) ^*^Resistance Exercise + ONS (n = 9)	ONS (n = 13)	^*^AE ± NS: 24 (4, 36) ^*^RE ± NS: 19 (8, 36)	28 (8, 48)	^*^Aerobic exercise: 20–30 min of pedaling stationary bike during the first 2 h of hemodialysis ^*^Resistance Exercise: 4 sets of 20 repetitions of resistance exercise for 40 min during the first 2 h of hemodialysis	1 can of ONS (including water, maltodextrin, canola oil, lactalbumin, ascorbic acid, and citric acid as an antioxidant) (480 kcal, 20 gr protein, 20 gr lipids, and 56 gr carbohydrates)	3
Martin-Alemañy et al. (30)	Mexico	RCT	Hemodialysis	34	24	(10) / (14)	One group: Combination of Aerobic and Resistance Exercise + ONS (n = 10)	ONS (n = 14)	33 ± 19	61 ± 43	30 min of cycling +4 sets of 20 repetitions of resistance exercise	2 cans of ONS Each can (434 kcal, 19.2 gr protein, and 22.8 gr lipids)	6

RCT, randomized controlled trial; EX; exercise; NS, nutritional support; RE, resistance exercise; AE, aerobic exercise; PEW, protein-energy wasting; IDON, intradialytic oral nutritional supplementation; ONC, oral nutritional complements; IDPN, intradialytic parenteral nutrition; ONS, oral nutritional supplementation; FOSs, fructooligosaccharides; NR, not reported.

* Dialysis Vintage is expressed as absolute number, mean ± SD or median (interquartile range).

### 3.2 Exercise plus nutritional support versus nutritional support

Eligible studies, including two pilot studies, compared the effects of exercise combined with nutritional support to nutritional support alone on biochemical markers of nutrition (albumin, C-reactive protein (CRP), and creatinine levels), weight, BMI, fat mass percentage (FM%), muscle mass and muscle strength in hemodialysis patients (25–30). The intervention group underwent both exercise and nutritional support, while the control group received only nutritional support.

#### 3.2.1 Biochemical markers of nutrition

Four studies [Dong et al. (25); Martin-Alemañy et al. (27); Jeong et al. (28); and Martin-Alemañy et al. (29)] found no statistically significant differences in albumin levels between the intervention and control groups. Similarly, three studies [Dong et al. (25); Jeong et al. (28); and Martin-Alemañy et al. (29)] observed no statistically significant intergroup differences in CRP levels. Likewise, three studies [Dong et al. (25); Martin-Alemañy et al. (27); and Martin-Alemañy et al. (29)] also revealed no statistically significant differences in creatinine levels between the groups. These results were supported by pilot studies conducted by Hristea et al. (26) and Martin-Alemañy et al. (30), which reported no significant intergroup differences in albumin, CRP, and creatinine levels. The changes in biochemical markers of nutrition are detailed in Table 4.

**Table 4:** “Biochemical Markers of Nutrition” changes.

Study	Variables	Intervention group (EX + NS) baseline		Intervention group (EX + NS) final		*p*-value^*^		Control group (NS) baseline	Control group (NS) final	*p*-value^*^	*p*-value^+^
Dong et al. (25)	^‡^Alb (g/dl)	4.07 ± 0.28		4.15 ± 0.44		> 0.05		4.18 ± 0.3	4.21 ± 0.22	> 0.05	> 0.05
	CRP (mg/l)	4.3 (1.9, 13.3)		2.6 (1.3, 8.3)		> 0.05		3.9 (1.0, 12.0)	6.9 (5.9, 12.4)	> 0.05	> 0.05
	Creatinine (mg/dl)	9.3 ± 2.5		10.2 ± 2.5		> 0.05		10.9 ± 2.3	11.7 ± 2.5	> 0.05	> 0.05
Hristea et al. (26)	^‡^Alb (g/dl)	3.83 ± 0.28		3.93 ± 0.25		>0.05		3.96 ± 0.37	3.91 ± 0.36	>0.05	>0.05
	CRP (mg/l)	5.71 ± 8.16		1.75 ± 1.62		>0.05		6.22 ± 5.78	4.99 ± 5.96	>0.05	>0.05
	^+^Creatinine (mg/dl)	5.54 ± 2.21		5.48 ± 1.62		>0.05		7.79 ± 2.04	8.2 ± 2.01	>0.05	>0.05
Martin-Alemañy et al. (27)	Alb (g/dl)	3.3 ± 0.25		3.7 ± 0 0.33		<0.001		3.5 ± 0.29	3.7 ± 0.35	<0.001	>0.05
	Creatinine (mg/dl)	13.9 ± 4.9		12.4 ± 4.2		>0.05		15.7 ± 5	13.7 ± 4.8	>0.05	>0.05
Jeong et al. (28)	Alb (g/dl)	3.92 ± 0.37		3.93 ± 0.51		>0.05		4.00 ± 0.35	4.01 ± 0.31	> 0.05	0.71
	CRP (mg/l)	15.2 ± 14.1		13.17 ± 11.8		>0.05		18.1 ± 20.9	11.36 ± 7.76	> 0.05	0.40
Martin-Alemañy et al. (30)	Alb (g/dl)	4.2 ± 0.53		4.2 ± 0.29		0.849		4.3 ± 0.41	4.3 ± 0.47	0.390	0.396
	CRP (mg/l)	4.5 (1.2, 12.8)		3.3 (2.9, 9)		0.594		5.6 (2.8, 8.9)	4.1 (2,7.3)	0.638	0.781
	Creatinine (mg/dl)	13.3 ± 3.5		13.5 ± 2.2		0.873		13.3 ± 2.8	11.4 ± 4.4	0.049	0.207
Martin-Alemañy et al. (29)		RE + NS (baseline)	AE + NS (baseline)	RE + NS (final)	AE + NS (final)	RE + NS	AE + NS				
	Alb (g/dl)	3.8 ± 0.52	3.6 ± 0.32	3.8 ± 0.44	3.4 ± 0.55	0.037	< 0.05	3.8 ± 0.47	3.9 ± 0.40	0.028	0.423
	CRP (mg/l)	3.4 (2.6, 9.2)	7.2 (3.3, 12.6)	6.2 (3.8, 9.3)	5.4 (2.8, 17.1)	0.110	0.784	3.5 (2.4, 4.4)	3.4 (1.9, 6.1)	0.834	0.441
	Creatinine (mg/dl)	12.6 ± 3.1	12.6 ± 3.3	11.9 ± 3.9	11.7 ± 3.6	0.095	0.037	14 ± 3.5	12.3 ± 3.4	0.078	0.732

Alb, albumin; CRP, C - reactive protein; EX; exercise, NS, nutritional support; RE, resistance exercise; AE, aerobic exercise; variables displayed as mean ± SD or median (first and third quartiles). *p*-value^*^ compare intragroup differences. *p*-value + compare intergroup differences. *p*-value < 0.05 indicates statistical significance.

‡ In Dong et al. (25) and Hristea et al. (26), albumin was reported in mg/dl, which was then converted to g/dl.

+ In Hristea et al. (26), Serum Creatinine was reported in umol/l, which was then converted to mg/dl.

#### 3.2.2 Anthropometry and body composition

##### 3.2.2.1 Body weight

Three studies [Dong et al. (25); Martin-Alemañy et al. (27); and Martin-Alemañy et al. (29)] indicated no statistically significant differences in body weight between the intervention and control groups. Similarly, a pilot study by Martin-Alemañy et al. (30) also revealed no significant intergroup differences in body weight. The changes in body weight are displayed in Table 5.

**Table 5:** “Anthropometry and Body Composition” assessment.

Study	Variables	Intervention group (EX + NS) baseline		Intervention group (EX + NS) final		*p*-value^*^		Control group (NS) baseline	Control group (NS) final	*p*-value^*^	*p*-value+
Dong et al. (25)	weight (kg)	75.8 ± 15.1		74.6 ± 12.7		>0.05		84.2 ± 17.3	86.2 ± 20.7	>0.05	>0.05
	BMI (kg/m^2^)	27.5 ± 6.3		26.8 ± 4.3		>0.05		29.1 ± 6.4	29.3 ± 6.8	>0.05	>0.05
	FM%	21.2 ± 9.5		19.9 ± 8.0		>0.05		19.4 ± 8.7	17.1 ± 8.0	>0.05	>0.05
Hristea et al. (26)	BMI (kg/m^2^)	20.51 ± 3.53		20.89 ± 1.19		> 0.1		20.81 ± 2.76	20.93 ± 2.57	> 0.1	> 0.7
Jeong et al. (28)	BMI (kg/m^2^)	31.9 ± 8.3		33.9 ± 10.9		> 0.05		30.6 ± 7.1	31.5 ± 7.4	> 0.05	0.64
	FM%	31.3 ± 10.1		30.9 ± 11.3		> 0.05		32.2 ± 9.9	32.4 ± 10.5	> 0.05	0.15
Martin-Alemañy et al. (27)	weight (kg)	51 (46, 56.7)		51.5 (46.5, 58)		< 0.05		46.5 (43, 52)	48.5 (45, 54)	< 0.05	> 0.05
	BMI (kg/m^2^)	20.4 (19.4, 23)		20.7 (19.6, 23.6)		< 0.05		21 (18.3, 22.1)	21.3 (20.1, 22.2)	< 0.05	> 0.05
	FM%	20 ± 8.6		20.3 ± 9		< 0.05		17 ± 6.5	17.6 ± 6.5	< 0.05	> 0.05
	MAC (cm)	23.5 (22, 27.3)		24.7 (23, 27.5)		< 0.05		24 (21.3, 26)	24.5 (23, 27)	< 0.05	> 0.05
	AMC (mm)	211.2 (191.3, 245)		217.4 (200.6, 241.7)		< 0.05		205 (192, 238.6)	220 (202.4, 244.3)	< 0.05	> 0.05
	AMA (cm^2^)	32 ± 12.8		29.8 ± 9.7		< 0.05		28.5 ± 11.49	29.7 ± 7.5	< 0.05	> 0.05
	Triceps skinfold thickness (mm)	9.1 ± 5.3		9.5 ± 5.4		< 0.05		7.7 ± 3.4	8.1 ± 3.6	< 0.05	> 0.05
Martin-Alemañy et al. (30)	weight (kg)	56.2 ± 8.8		58.2 ± 9.2		0.001		54.7 ± 7.4	55.8 ± 6.7	0.014	0.462
	FM%	21.1 ± 7		22.9 ± 7.9		0.046		23 ± 8.4	23.8 ± 8.2	0.311	0.793
	MAC (cm)	27.1 ± 3.5		26.9 ± 3.1		0.778		27 ± 3.1	26 ± 3	0.151	0.770
	AMC (mm)	228 (209–257)		226 (207–246)		0.508		230 (213–249)	220 (207–238)	0.084	0.886
	AMA (cm^2^)	36 ± 9.8		34.7 ± 9.2		0.544		37 ± 8.8	33.9 ± 9.1	0.097	0.838
	Triceps skinfold thickness (mm)	13 ± 5.1		13.7 ± 5.2		0.066		12.8 ± 4.6	13.1 ± 5.2	0.537	0.798
Martin-Alemañy et al. (29)		RE + NS (baseline)	AE + NS (baseline)	RE + NS (final)	AE + NS (final)	RE + NS	AE + NS				
	Weight (kg)	53.3 ± 6	52.2 ± 8.5	54.9 ± 5.1	52.8 ± 8.3	0.006	0.097	52 ± 9.7	53 ± 9.3	0.032	0.216
	BMI (kg/m^2^)	21.4 ± 1.2	20.8 ± 2.8	22.1 ± 1	21 ± 2.6	0.006	0.123	19.4 ± 1.6	19.8 ± 1.7	0.035	0.209
	FM%	21.2 ± 6.1	19.6 ± 7.5	22.4 ± 6.2	20.3 ± 7.7	0.031	0.316	14.3 ± 5.7	15.1 ± 5.6	0.082	0.797
	MAC (cm)	25.8 ± 2.1	25.3 ± 2.8	26.2 ± 2.1	25.5 ± 2.7	0.209	0.590	24.1 ± 2.3	24.4 ± 2.1	0.179	0.843
	AMC (mm)	224.4 ± 23.1	220.6 ± 24.7	225.1 ± 23.4	219 ± 25	0.839	0.666	217.9 ± 27.6	219.9 ± 24	0.410	0.685
	AMA (cm^2^)	33.9 ± 8.5	32.7 ± 8.8	34.2 ± 8.6	32.4 ± 8.8	0.821	0.684	31.8 ± 9.7	34.4 ± 8.4	0.500	0.752
	Triceps skinfold thickness (mm)	10.8 ± 3.2	10.5 ± 4.3	11.7 ± 3.2	11.2 ± 4.6	0.035	0.222	7.3 ± 3	7.8 ± 2.6	0.139	0.780

BMI, body mass index; FM%, fat mass percentage; MAC, mid-arm circumference; AMC, arm muscle circumference; AMA, arm muscle area; EX; exercise, NS, nutritional support; RE, resistance exercise; AE, aerobic exercise; variables displayed as mean ± SD or median (first and third quartiles). *p*-value^*^ compare intragroup differences. *p*-value + compare intergroup differences. *p*-value < 0.05 indicates statistical significance.

##### 3.2.2.2 BMI and FM %

Four studies [Dong et al. (25); Martin-Alemañy et al. (27); Jeong et al. (28); and Martin-Alemañy et al. (29)] reported no statistically significant differences in BMI and FM % between the groups. Two pilot studies also confirmed these findings, which Hristea et al. (26) found no significant intergroup differences in BMI and Martin-Alemañy et al. (30) found no significant intergroup differences in FM%. The changes in BMI and FM% are reported in Table 5.

##### 3.2.2.3 Muscle mass

Two studies [Martin-Alemañy et al. (27); and Martin-Alemañy et al. (29)] assessed muscle mass using mid-arm circumference (MAC), arm muscle circumference (AMC), arm muscle area (AMA), and triceps skinfold thickness. They both reported no statistically significant differences in muscle mass between the intervention and control groups. Likewise, a pilot study by Martin-Alemañy et al. (30) observed no significant intergroup differences in muscle mass. The detailed results regarding muscle mass are available in Table 5.

##### 3.2.2.4 Muscle strength

Four studies [Dong et al. (25); Martin-Alemañy et al. (27); Jeong et al. (28); and Martin-Alemañy et al. (29)] used handgrip strength (HGS), one Repetition Maximum (1-RM), knee extension maximal strength, and leg maximal flexion force as the measures of muscle strength for comparison. They all indicated no statistically significant differences in muscle strength between the intervention and control groups. Additionally, pilot studies by Hristea et al. (26) and Martin-Alemañy et al. (30) also reported no significant intergroup differences in muscle strength (26, 30). The detailed results for muscle strength assessment are provided in Table 6.

**Table 6:** “Muscle strength” assessment.

Study	Variables	Intervention group (EX + NS) baseline		Intervention group (EX + NS) final		*p*-value^*^		Control group (NS) baseline	Control group (NS) final	*p*-value^*^	*p*-value+
Dong et al. (25)	1-RM (lb)	459 ± 117		582 ± 147		> 0.05		475 ± 175	527 ± 139	> 0.05	0.12
Hristea et al. (26)	Knee extension maximal strength (kg)	10.22 ± 4.95		10.56 ± 3.49		> 0.05		9.97 ± 4.41	7.87 ± 2.19	0.02	> 0.05
Martin-Alemañy et al. (27)	HGS (kg)	20 (16, 24.5)		22 (17.5, 31)		< 0.05		16 (10, 24)	20.6 (15–27)	< 0.05	> 0.05
Jeong et al. (28)	Leg extension (ft-lb)	80.9 ± 32.8		85.7 ± 36.6		<0.05		73.9 ± 40.2	77.4 ± 38.7	<0.05	0.75
	Leg flexion (ft-lb)	36.5 ± 18.9		45.6 ± 34.8		<0.05		37.8 ± 20.4	38.9 ± 22.1	>0.05	0.24
Martin-Alemañy et al. (30)	HGS (kg)	21.56 (13.72, 39.21)		23.52 (17.15, 40.19)		0.016		21.56 (11.27, 36.27)	23.52 (12.25, 36.76)	0.014	0.872
Martin-Alemañy et al. (29)	HGS (kg)	RE + NS (baseline)	AE + NS (baseline)	RE + NS (final)	AE + NS (final)	RE + NS	AE + NS				
		22.9 (16.53, 29.19)	21.2 (17.89, 24.43)	25.11 (17.8, 32.34)	23.16 (19.5, 26.73)	< 0.05	< 0.05	26.3 (20.95, 31.75)	26.73 (22.14, 31.32)	0.000	> 0.05

1-RM, one repetition maximum; HGS, handgrip strength; EX; exercise, NS, nutritional support; RE, resistance exercise; AE, aerobic exercise; variables displayed as median with 95% confidence interval or mean ± SD. [median (first and third quartiles)]. *p*-value^*^ compare intragroup differences. *p*-value + compare intergroup differences. *p*-value < 0.05 indicates statistical significance.

## 4 Discussion

We conducted this systematic review to investigate whether the addition of exercise to nutritional support provides further benefits for biochemical indicators of nutritional status and body composition in hemodialysis patients. Given the potential advantages of exercise and nutritional care in this population, exploring the combined effects of these approaches might contribute to improving the overall well-being of patients.

### 4.1 Main findings

This review revealed that combining an exercise program with nutritional support does not produce synergistic effects on biochemical markers of nutrition, anthropometry, and body composition in hemodialysis patients. However, when these two interventions are examined separately, studies suggest that both exercise and nutritional support may induce anabolic effects. For instance, adequate nutritional support can enhance nutritional markers and improve protein homeostasis (31). Protein intake—especially essential amino acids (EAAs)—activates the mTORC1 pathway in skeletal muscle cells. This activation results in phosphorylating two key downstream targets: eukaryotic translation initiation factor 4E-binding protein 1 (4E-BP1) and ribosomal protein S6 kinase beta-1 (S6K1). This process promotes protein synthesis by facilitating the assembly of the translation initiation complex and recruiting ribosomes to mRNA (32). Additionally, exercise alone may help prevent muscle atrophy by inhibiting apoptotic processes and protein degradation (33). Exercise also stimulates muscle protein synthesis by activating mTORC1 through the Akt/PKB pathway. Akt/PKB further phosphorylates and inhibits glycogen synthase kinase three beta (GSK-3β), a negative regulator of mTORC1 (32). Given the ability of exercise to enhance the sensitivity of muscle cells to amino acids (32), we hypothesized that a combination of protein ingestion and physical exercise may offer additional benefits for hemodialysis patients. However, comparing changes between groups receiving both interventions and those receiving only nutritional support did not confirm our hypothesis. Although this systematic review was conducted rigorously and considered all available data, the findings should be interpreted cautiously due to concerns about the quality of the included studies.

### 4.2 Overview of the studies reviewed

This systematic review included six randomized controlled trials that examined the effects of adding exercise to nutritional support compared to receiving nutritional support alone (25–30). The interventions spanned from 3 to 12 months and involved supervised exercise programs (such as resistance, aerobic, or a combination) along with oral nutritional supplements (ONS) or intradialytic parenteral nutrition (IDPN). In our review of the included trials, we observed that some studies, emphasizing a resistance training program, demonstrated significant improvements in albumin and some body composition indices across all participants (27, 29). This may suggest the potential of resistance exercise to ameliorate muscle wasting; however, the findings also indicated no statistically significant differences in nutritional status and body composition between the groups receiving both interventions and those receiving only nutritional support. Several factors may explain the lack of significant impact of combining exercise with nutritional support. For instance, the studies provided data on the caloric or protein content of the nutritional support, but the adequacy of these values in relation to the patients’ overall dietary intake remains uncertain, making it difficult to understand the effectiveness of the exercise programs in improving body composition (25–30). Some studies did not also report sample size calculations, leaving the power to detect differences unclear (26–28). Additionally, high dropout rates may have influenced the results (26, 28), and there were issues with adherence reporting (25, 26, 28, 30) and the short duration of some interventions (27, 29). Furthermore, the inclusion of younger patients with good nutritional status or physical performance may have restricted the ability to detect clear benefits (25, 29, 30). Overall, due to the insufficient power, we could not conclusively determine the effects of combined nutritional and exercise interventions in hemodialysis patients.

### 4.3 Strengths and limitations

The present literature review consists of some strengths and limitations. This is the first systematic review which evaluates the potential effects of adding exercise to nutritional support in hemodialysis patients. We included studies with a control group receiving nutritional support to observe the effects of exercise clearly. Our literature search was also comprehensive, and the included articles were relatively new and well-designed. However, most studies had a small number of patients and a short duration of follow-up. High heterogeneity, failure to find the source of the heterogeneity, and the risk of bias in some factors should be considered limitations. Additionally, two of the included studies were pilot studies, so their findings may not be conclusive due to the low power. Moreover, we did not assess the risk of publication bias due to the inability of performing a meta-analysis, which is considered a methodological limitation of this review.

## 5 Conclusion

In conclusion, our findings suggest that adding exercise may not enhance the anabolic effects of nutritional support on anthropometry, body composition, and nutritional status in hemodialysis patients. However, these conclusions are not definitive due to the limited statistical power and heterogeneous nature of the existing studies. The low quality of these studies warrants cautious interpretation of the results. Therefore, further high-quality clinical trials with larger sample sizes, longer durations, and more robust designs are recommended to provide more accurate and comprehensive insights into the potential benefits of combining exercise with nutritional support in hemodialysis patients.

## Data Availability

The original contributions presented in the study are included in the article/Supplementary material, further inquiries can be directed to the corresponding author.

## References

[1] Agarwal R . Defining end-stage renal disease in clinical trials: a framework for adjudication. Nephrol Dial Transplant. (2016) 31:864–7. doi: 10.1093/ndt/gfv289, PMID: 26264780

[2] Liyanage T Ninomiya T Jha V Neal B Patrice HM Okpechi I . Worldwide access to treatment for end-stage kidney disease: a systematic review. Lancet. (2015) 385:1975–82. doi: 10.1016/S0140-6736(14)61601-9, PMID: 25777665

[3] Pecoits-Filho R Okpechi IG Donner J-A Harris DC Aljubori HM Bello AK . Capturing and monitoring global differences in untreated and treated end-stage kidney disease, kidney replacement therapy modality, and outcomes. Kidney Int Suppl. (2020) 10:e3–9. doi: 10.1016/j.kisu.2019.11.001, PMID: 32149004 PMC7031690

[4] Bello AK Levin A Tonelli M Okpechi IG Feehally J Harris D . Assessment of global kidney health care status. JAMA. (2017) 317:1864–81. doi: 10.1001/jama.2017.4046, PMID: 28430830 PMC5470418

[5] Bello AK Levin A Lunney M Osman MA Ye F Ashuntantang GE . Status of Care for end Stage Kidney Disease in countries and regions worldwide: international cross sectional survey. BMJ. (2019) 367:l5873. doi: 10.1136/bmj.l5873, PMID: 31672760

[6] Oliveira EA Zheng R Carter CE Mak RH. Cachexia/protein energy wasting syndrome in CKD: causation and treatment. Semin Dial. (2019) 32:493–9. doi: 10.1111/sdi.12832, PMID: 31286575

[7] Lodebo BT Shah A Kopple JD. Is it important to prevent and treat protein-energy wasting in chronic kidney disease and chronic Dialysis patients? J Ren Nutr. (2018) 28:369–79. doi: 10.1053/j.jrn.2018.04.002, PMID: 30057212

[8] Fouque D Kalantar-Zadeh K Kopple J Cano N Chauveau P Cuppari L . A proposed nomenclature and diagnostic criteria for protein–energy wasting in acute and chronic kidney disease. Kidney Int. (2008) 73:391–8. doi: 10.1038/sj.ki.5002585, PMID: 18094682

[9] Kalantar-Zadeh K Kilpatrick RD Kuwae N McAllister CJ Alcorn H Jr Kopple JD . Revisiting mortality predictability of serum albumin in the Dialysis population: time dependency, longitudinal changes and population-attributable fraction. Nephrol Dial Transplant. (2005) 20:1880–8. doi: 10.1093/ndt/gfh941, PMID: 15956056

[10] Ikizler TA Wingard RL Harvell J Shyr Y Hakim RM. Association of Morbidity with markers of nutrition and inflammation in chronic hemodialysis patients: a prospective study. Kidney Int. (1999) 55:1945–51. doi: 10.1046/j.1523-1755.1999.00410.x, PMID: 10231458

[11] Clyne N Jogestrand T Lins LE Pehrsson SK Ekelund LG. Factors limiting physical working capacity in Predialytic Uraemic patients. Acta Med Scand. (1987) 222:183–90. doi: 10.1111/j.0954-6820.1987.tb10657.x, PMID: 3673671

[12] Cheema BSB Fiatarone Singh MA. Exercise training in patients receiving maintenance hemodialysis: a systematic review of clinical trials. Am J Nephrol. (2005) 25:352–64. doi: 10.1159/000087184, PMID: 16088076

[13] Clapp EL Bevington A Smith AC. Exercise for children with chronic kidney disease and end-stage renal disease. Pediatr Nephrol. (2012) 27:165–72. doi: 10.1007/s00467-010-1753-1, PMID: 21229267

[14] Kosmadakis GC John SG Clapp EL Viana JL Smith AC Bishop NC . Benefits of regular walking exercise in advanced pre-Dialysis chronic kidney disease. Nephrol Dial Transplant. (2012) 27:997–1004. doi: 10.1093/ndt/gfr364, PMID: 21795756

[15] Levin A Ahmed SB Carrero JJ Foster B Francis A Hall RK . Executive summary of the Kdigo 2024 clinical practice guideline for the evaluation and Management of Chronic Kidney Disease: known knowns and known unknowns. Kidney Int. (2024) 105:684–701. doi: 10.1016/j.kint.2023.10.016, PMID: 38519239

[16] Olvera-Soto MG Valdez-Ortiz R Alvarenga JCL de los Ángeles Espinosa-Cuevas M. Effect of resistance exercises on the indicators of muscle reserves and handgrip strength in adult patients on hemodialysis. J Ren Nutr. (2016) 26:53–60. doi: 10.1053/j.jrn.2015.06.006, PMID: 26264173

[17] Torres E Aragoncillo I Moreno J Vega A Abad S García-Prieto A . Exercise training during hemodialysis sessions: physical and biochemical benefits. Ther Apher Dial. (2020) 24:648–54. doi: 10.1111/1744-9987.13469, PMID: 31886624

[18] Liao M-T Liu W-C Lin F-H Huang C-F Chen S-Y Liu C-C . Intradialytic aerobic cycling exercise alleviates inflammation and improves endothelial progenitor cell count and bone density in hemodialysis patients. Medicine. (2016) 95:e4134. doi: 10.1097/MD.0000000000004134, PMID: 27399127 PMC5058856

[19] Rattanasompattikul M Molnar MZ Lee ML Dukkipati R Bross R Jing J . Anti-inflammatory and anti-oxidative nutrition in Hypoalbuminemic Dialysis patients (Aionid) study: results of the pilot-feasibility, double-blind, randomized, placebo-controlled trial. J Cachexia Sarcopenia Muscle. (2013) 4:247–57. doi: 10.1007/s13539-013-0115-9, PMID: 24052226 PMC3830006

[20] Stratton RJ Bircher G Fouque D Stenvinkel P De Mutsert R Engfer M . Multinutrient Oral supplements and tube feeding in maintenance Dialysis: a systematic review and Meta-analysis. Am J Kidney Dis. (2005) 46:387–405. doi: 10.1053/j.ajkd.2005.04.036, PMID: 16129200

[21] Qin A Tan J Hu W Liu Y Chen L Tang Y . Oral energy supplementation improves nutritional status in hemodialysis patients with protein–energy wasting: a pilot study. Front Pharmacol. (2022) 13:839803. doi: 10.3389/fphar.2022.839803, PMID: 36339616 PMC9633655

[22] Battaglia Y Baciga F Bulighin F Amicone M Mosconi G Storari A . Physical activity and exercise in chronic kidney disease: consensus statements from the physical exercise working Group of the Italian Society of nephrology. J Nephrol. (2024) 37:–1765. doi: 10.1007/s40620-024-02049-9, PMID: 39269600 PMC11519309

[23] Page MJ McKenzie JE Bossuyt PM Boutron I Hoffmann TC Mulrow CD . The Prisma 2020 statement: an updated guideline for reporting systematic reviews. BMJ. (2021) 372:n71. doi: 10.1136/bmj.n71, PMID: 33782057 PMC8005924

[24] Sterne JAC Savović J Page MJ Elbers RG Blencowe NS Boutron I . Rob 2: a revised tool for assessing risk of Bias in randomised trials. BMJ. (2019) 366:l4898. doi: 10.1136/bmj.l4898, PMID: 31462531

[25] Dong J Sundell MB Pupim LB Wu P Shintani A Ikizler TA. The effect of resistance exercise to augment long-term benefits of intradialytic Oral nutritional supplementation in chronic hemodialysis patients. J Ren Nutr. (2011) 21:149–59. doi: 10.1053/j.jrn.2010.03.004, PMID: 20580251 PMC2947559

[26] Hristea D Deschamps T Paris A Lefrançois G Collet V Savoiu C . Combining intra-dialytic exercise and nutritional supplementation in malnourished older Haemodialysis patients: towards better quality of life and autonomy. Nephrology (Carlton). (2016) 21:785–90. doi: 10.1111/nep.12752, PMID: 26890997

[27] Martin-Alemañy G Valdez-Ortiz R Olvera-Soto G Gomez-Guerrero I Aguire-Esquivel G Cantu-Quintanilla G . The effects of resistance exercise and Oral nutritional supplementation during hemodialysis on indicators of nutritional status and quality of life. Nephrol Dial Transplant. (2016) 31:1712–20. doi: 10.1093/ndt/gfw297, PMID: 27510532

[28] Jeong JH Biruete A Tomayko EJ Wu PT Fitschen P Chung HR . Results from the randomized controlled Ihope trial suggest no effects of Oral protein supplementation and exercise training on physical function in hemodialysis patients. Kidney Int. (2019) 96:777–86. doi: 10.1016/j.kint.2019.03.018, PMID: 31200945 PMC6708720

[29] Martin-Alemañy G Espinosa-Cuevas M Pérez-Navarro M Wilund KR Miranda-Alatriste P Cortés-Pérez M . Effect of Oral nutritional supplementation with and without exercise on nutritional status and physical function of adult hemodialysis patients: a parallel controlled clinical trial (Avante-Hemo study). J Ren Nutr. (2020) 30:126–36. doi: 10.1053/j.jrn.2019.06.010, PMID: 31607547

[30] Martin-Alemañy G Perez-Navarro M Wilund KR García-Villalobos G Gómez-Guerrero I Cantú-Quintanilla G . Effect of intradialytic Oral nutritional supplementation with or without exercise improves muscle mass quality and physical function in hemodialysis patients: a pilot study. Nutrients. (2022) 14:2946. doi: 10.3390/nu14142946, PMID: 35889902 PMC9323958

[31] Dong J Ikizler TA. New insights into the role of anabolic interventions in Dialysis patients with protein energy wasting. Curr Opin Nephrol Hypertens. (2009) 18:469–75. doi: 10.1097/MNH.0b013e328331489d, PMID: 19713839 PMC2891019

[32] Garibotto G Saio M Aimasso F Russo E Picciotto D Viazzi F . How to overcome anabolic resistance in Dialysis-treated patients? Front Nutr. (2021) 8:701386. doi: 10.3389/fnut.2021.701386, PMID: 34458305 PMC8387577

[33] Deligiannis A D'Alessandro C Cupisti A. Exercise training in Dialysis patients: impact on cardiovascular and skeletal muscle health. Clin Kidney J. (2021) 14:ii25–33. doi: 10.1093/ckj/sfaa273, PMID: 33981417 PMC8101623

[34] Magnard J Deschamps T Cornu C Paris A Hristea D. Effects of a six-month intradialytic physical activity program and adequate nutritional support on protein-energy wasting, physical functioning and quality of life in chronic hemodialysis patients: Actinut study protocol for a randomised controlled trial. BMC Nephrol. (2013) 14:259. doi: 10.1186/1471-2369-14-259, PMID: 24279747 PMC4222262

