# Supplementary material for: Does exercise enhance the benefits of nutritional support on the biochemical markers of nutrition, anthropometry, and body composition in hemodialysis patients? A systematic review
Source: Front Nutr. 2024 Nov 29;11:1471455. doi: 10.3389/fnut.2024.1471455 (PMC11637855; doi:10.3389/fnut.2024.1471455)
Supplement: Supplementary file 1 [file Table_1.DOCX]

Supplementary Material

**Supplementary table 1. Search Strategy**

| **Terms** | |
| --- | --- |
| **Intervention** | **Outcomes** |
| "Nutritional support" OR "Nutritional supplementation" OR "Oral nutritional supplementation" OR "Nutritional intervention" OR "Nutritional therapy" OR "Enteral nutrition" OR "Parenteral nutrition"  AND  "Exercise" OR "Physical fitness" OR "Sports" OR "Exercise therapy" OR "Physical Activity" OR "Exercise training" OR "Physical exercise" | "Body composition" OR "Body fat distribution" OR "Body fat percentage" OR "Lean body mass" OR LBM OR "Body weights and measures" OR "Body mass index" OR BMI OR "Anthropometry" OR "Skinfold thickness" OR "Waist-Hip ratio"  OR  "Biomarkers of nutritional status" OR "Nutritional status indicators" OR "Nutritional status" OR "Nutritional assessment" OR "Nutritional biomarkers" OR "Malnutrition" OR "Biochemical indicators" OR "Biochemical parameters" |

**Supplementary table 2. Search Strategy**

| **Database** | **n** |
| --- | --- |
| **PubMed:**  (((("Enteral Nutrition"[Mesh] OR "Nutritional Support"[Mesh]) OR ("Nutritional support"[Title/Abstract] OR "Nutritional supplementation"[Title/Abstract] OR "Oral nutritional supplementation"[Title/Abstract] OR "Nutritional intervention"[Title/Abstract] OR "Nutritional therapy"[Title/Abstract] OR "Enteral nutrition"[Title/Abstract] OR "Parenteral nutrition"[Title/Abstract])) AND (("Exercise Therapy"[Mesh] OR "Exercise"[Mesh]) OR ("Exercise"[Title/Abstract] OR "Physical fitness"[Title/Abstract] OR "Sports"[Title/Abstract] OR "Exercise therapy"[Title/Abstract] OR "Physical Activity"[Title/Abstract] OR "Exercise training"[Title/Abstract] OR "Physical exercise"[Title/Abstract]))) AND ((("Body Composition"[Mesh]) OR "Anthropometry"[Mesh]) OR ("Body composition" OR "Body fat distribution" OR "Body fat percentage" OR "Lean body mass" OR LBM OR "Body weights and measures" OR "Body mass index" OR BMI OR "Anthropometry" OR "Skinfold thickness" OR "Waist-Hip ratio"))) AND (("Nutritional Status"[Mesh] OR "Nutrition Assessment"[Mesh] OR "Malnutrition"[Mesh]) OR ("Biomarkers of nutritional status" OR "Nutritional status indicators" OR "Nutritional status" OR "Nutritional assessment" OR "Nutritional biomarkers" OR "Malnutrition" OR "Biochemical indicators" OR "Biochemical parameters")) | **195** |
| **Scopus:**  ( TITLE-ABS-KEY ( "Nutritional support" OR "Nutritional supplementation" OR "Oral nutritional supplementation" OR "Nutritional intervention" OR "Nutritional therapy" OR "Enteral nutrition" OR "Parenteral nutrition" ) AND TITLE-ABS-KEY ( "Exercise" OR "Physical fitness" OR "Sports" OR "Exercise therapy" OR "Physical Activity" OR "Exercise training" OR "Physical exercise" ) AND TITLE-ABS-KEY ( "Body composition" OR "Body fat distribution" OR "Body fat percentage" OR "Lean body mass" OR lbm OR "Body weights and measures" OR "Body mass index" OR bmi OR "Anthropometry" OR "Skinfold thickness" OR "Waist-Hip ratio" ) AND TITLE-ABS-KEY ( "Biomarkers of nutritional status" OR "Nutritional status indicators" OR "Nutritional status" OR "Nutritional assessment" OR "Nutritional biomarkers" OR "Malnutrition" OR "Biochemical indicators" OR "Biochemical parameters" ) ) | **509** |
| **Web of Science:**  "Nutritional support" OR "Nutritional supplementation" OR "Oral nutritional supplementation" OR "Nutritional intervention" OR "Nutritional therapy" OR "Enteral nutrition" OR "Parenteral nutrition" (Topic) AND "Exercise" OR "Physical fitness" OR "Sports" OR "Exercise therapy" OR "Physical Activity" OR "Exercise training" OR "Physical exercise" (Topic) AND "Body composition" OR "Body fat distribution" OR "Body fat percentage" OR "Lean body mass" OR LBM OR "Body weights and measures" OR "Body mass index" OR BMI OR "Anthropometry" OR "Skinfold thickness" OR "Waist-Hip ratio" (All Fields) AND "Biomarkers of nutritional status" OR "Nutritional status indicators" OR "Nutritional status" OR "Nutritional assessment" OR "Nutritional biomarkers" OR "Malnutrition" OR "Biochemical indicators" OR "Biochemical parameters" (All Fields) | **224** |
